# Direct Observation of Circularly Polarized Nonlinear Optical Activities in Chiral Hybrid Lead Halides

**DOI:** 10.1021/jacs.4c00619

**Published:** 2024-04-03

**Authors:** Sunhao Liu, Xiaoming Wang, Yixuan Dou, Qian Wang, Jiyoon Kim, Carla Slebodnick, Yanfa Yan, Lina Quan

**Affiliations:** † Department of Chemistry, Virginia Tech, Blacksburg, Virginia 24061, United States; ‡ Department of Materials and Science Engineering, Virginia Tech, Blacksburg, Virginia 24061, United States; § Department of Physics and Astronomy and Wright Center for Photovoltaics Innovation and Commercialization, The University of Toledo, Toledo, Ohio 43606, United States

## Abstract

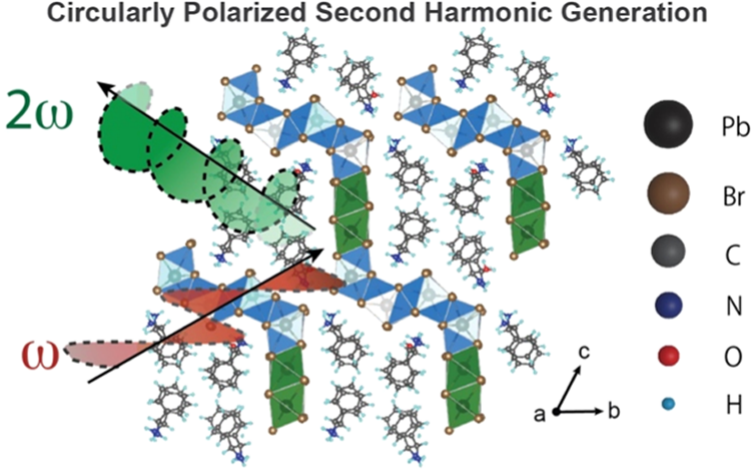

Circularly polarized light emission is a crucial application in imaging, sensing, and photonics. However, utilizing low-energy photons to excite materials, as opposed to high-energy light excitation, can facilitate deep-tissue imaging and sensing applications. The challenge lies in finding materials capable of directly generating circularly polarized nonlinear optical effects. In this study, we introduce a chiral hybrid lead halide (CHLH) material system, R/S-DPEDPb_3_Br_8_·H_2_O (DPED = 1,2-diphenylethylenediammonium), which can directly produce circularly polarized second harmonic generation (CP-SHG) through linearly polarized infrared light excitation, exhibiting a polarization efficiency as high as 37% at room temperature. To understand the spin relaxation mechanisms behind the high polarization efficiency, we utilized two models, so-called D’yakonov–Perel’ (DP) and Bir–Aronov–Pikus (BAP) mechanisms. The unique zigzag inorganic frameworks within the hybrid structure are believed to reduce the dielectric confinement and exciton binding energy, thus enhancing spin polarization, especially in regions with a high excitation pump fluence based on the DP mechanism. In the case of low excitation pump fluence, the BAP mechanism dominates, as evidenced by the observed decrease in the polarization ratio from CP-SHG measurement. Using density functional theory analysis, we elucidate how the distinctive 8-coordination environment of lead bromide building blocks effectively suppresses spin–orbit coupling at the conduction band minimum. This suppression significantly diminishes spin-splitting, thereby slowing the spin relaxation rate.

## Introduction

Nonlinear optical (NLO) materials possess important applications in the fields of imaging, photonics, and optoelectronics.^1−3^ Second harmonic generation (SHG) is a type of NLO process in which two photons of one frequency (ω) combine to generate a photon at twice the frequency (2ω) and is typically achieved with high-power, ultrafast infrared (IR) light excitation. Polarized SHG holds significant importance in biological imaging, which requires sensitivity to molecular alignment.^4−6^ However, the generation of circularly polarized SHG (CP-SHG) often relies on complex optical configurations that necessitate components such as quarter-wave plates. Direct generation of CP-SHG light continues to be challenging due to the limited availability of appropriate nonlinear chiral crystals. For example, two-dimensional transition-metal dichalcogenides (2D-TMDs) can generate CP-SHG light through the spin-valley effect and valley-dependent optical selection rules.^7,8^ Additionally, colloidal metal nanocrystals are capable of producing CP-SHG due to their anisotropic structures,^9^ which can be further enhanced through plasmon-resonant excitation.^10^ However, the synthesis of such materials often involves complicated processes and demanding conditions,^11,12^ which hinder progress in achieving effective polarization control in NLO devices.

Recently, an emerging class of materials that introduces chirality through a solution-processable approach has gained attention: chiral hybrid lead halide (CHLH) solids. This material family is considered promising for chiroptical and spintronic applications, attributed to their Rashba spin-splitting resulting from substantial spin–orbital coupling (SOC) induced by heavy elements and their noncentrosymmetric polar crystal structure.^13−16^ Circularly polarized photoluminescence can readily be achieved with CHLHs, eliminating the need for additional optical components.^17−19^ Furthermore, CHLHs exhibit NLO behavior due to lowering of the symmetry such that the materials are not only noncentrosymmetric but also chiral,^20−22^ thereby opening up new possibilities for advanced control over NLO polarization. However, the previously introduced CHLHs showed selective absorption of circularly polarized light and detected the resulting circularly polarized SHG signal to validate their chiral NLO properties. Therefore, it is crucial to advance the CHLHs capable of directly detecting CP-SHG light through excitation with linearly polarized light with low photon energy.

Herein, we used the chiral organic dication 1,2-diphenylethylenediammonium (DPED) to synthesize two-dimensional zigzag-structured lead bromide hybrid single crystals (R/S-DPEDPb_3_Br_8_·H_2_O) through a combination of organic template formation and oil–water interface growing method. The unique zigzag crystal structure reduced both quantum and dielectric confinement effects, primarily due to the short interlayer spacing, which potentially decreases excitonic binding energy. Remarkably, R/S-DPEDPb_3_Br_8_·H_2_O exhibited a strong SHG signal, showing an intensity 10 times greater than that of potassium dihydrogen phosphate (KDP) single crystals under identical measurement conditions. Additionally, CP-SHG was directly observed with 1064 nm linearly polarized near-infrared (NIR) light excitation, achieving a polarization ratio of up to 37%. The high polarization ratio can be attributed to a robust SOC in the valence band and a weak SOC in the conduction band, the anisotropic noncentrosymmetric polar structure governed by efficient chirality transfer as well as the reduced dielectric and quantum confinements.

Further analysis revealed that the governing mechanisms for CP-SHG were associated with D’yakonov–Perel’ (DP) and Bir–Aronov–Pikus (BAP) spin relaxation mechanisms. To complement these experimental findings, theoretical insights based on first-principles density functional theory (DFT) calculations were provided. These calculations confirmed the presence of a strong SOC in the valence band and a weak SOC within the conduction band, which contributed to the reduced spin relaxation process across both bands. This phenomenon increases the spin polarization, resulting in a high CP-SHG polarization ratio. Additionally, (R/S-DPED)Pb_3_Br_8_ also demonstrated a strong third harmonic generation (THG) signal, as confirmed by power-dependent and wavelength-dependent THG measurements. Both THG and SHG signals exhibited high laser damage thresholds, reaching up to 3.7 and 4.1 mJ/cm^2^, respectively. These NLO properties extend the potential applications of nonlinear polarized optical applications.

## Results and Discussion

**Figure 1:**
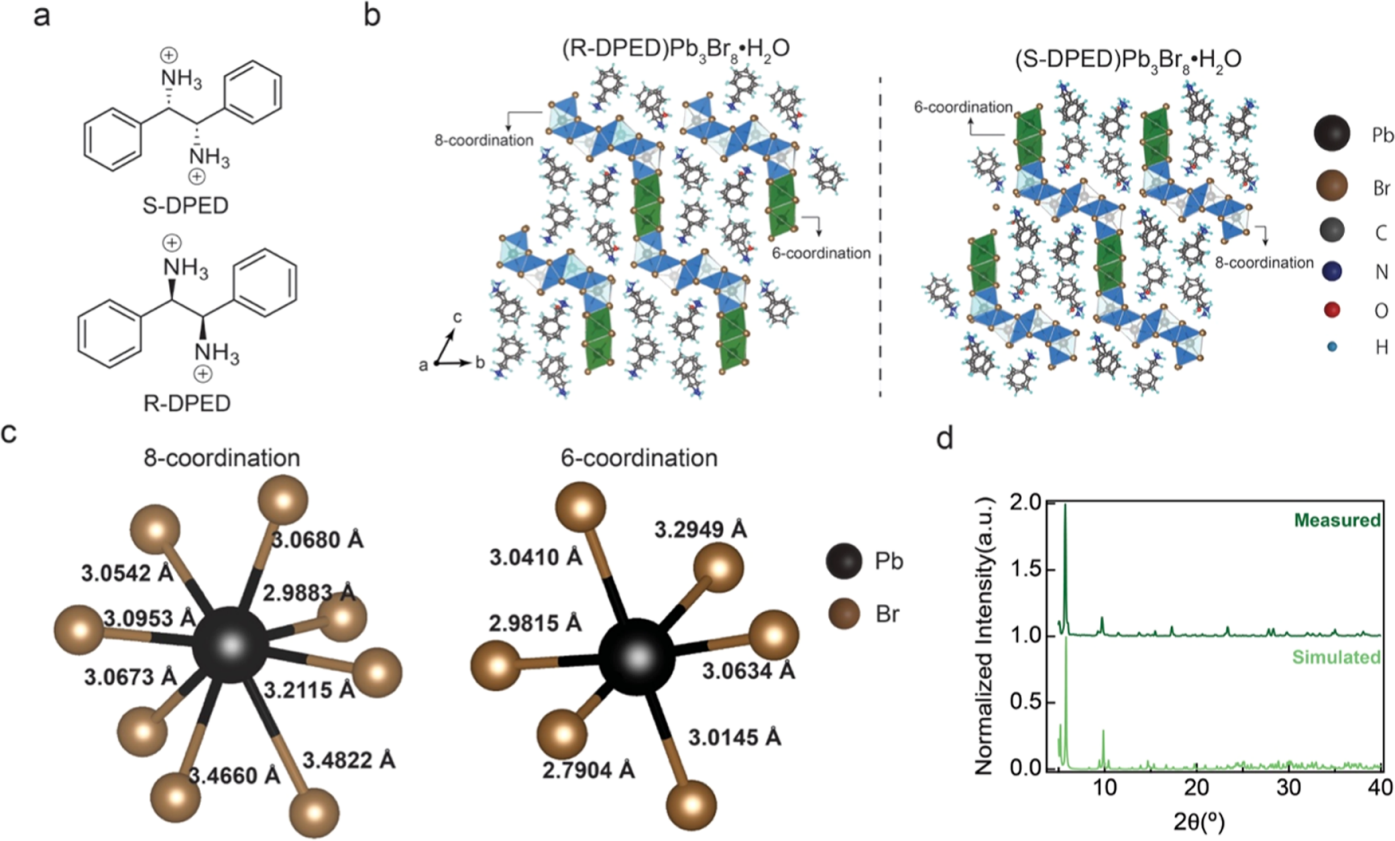
(a) Chemical structure of the organic spacer of DPED. (b) Crystal structures of R-DPEDPb_3_Br_8_·H_2_O (left) and S-DPEDPb_3_Br_8_·H_2_O (right). (c) 8-coordinate (left) or 6-coordinate (right) representations of two of the 12 unique Pb atoms in the Pb–Br framework labeled with the respective Pb–Br bond lengths. (d) Powder X-ray diffraction (PXRD) data of as-synthesized R-DPEDPb_3_Br_8_·H_2_O grind single crystals and simulated PXRD data from single-crystal XRD.

Figure 1a illustrates the molecular structure of R-1,2-diphenylethylenediammonium or S-1,2-diphenylethylenediammonium (R/S-DPED) organic spacer dications. The large size and steric effects of the organic spacer elevate the nucleation barrier in the single-crystal synthesis, thereby making it challenging for the formation of nucleation seeds.^23^ As the nucleation barrier energy at the solution surface is lower than that in the bulk solution, the possibility of crystal seed formation at the surface is higher than that in the solution.^24,25^ Thus, the water–oil interface crystallization method was used successfully to grow R/S-DPEDPb_3_Br_8_·H_2_O single crystals. The crystallization process unfolds into two distinct stages: nucleation and growth. Initially, at the water–oil interface, the hydrophilic ammonium cationic functional group exhibited stronger attraction to water molecules, driven by static coulomb interaction. Conversely, the lipophilic phenyl group is more attracted to the oil side. This results in the formation of oriented monolayers at the water–oil interface with the ammonium cationic functional group pointed down, which serves as the template to guide crystal growth. During the crystal growth stage, the Pb and Br ions diffuse toward the water–oil interface, establishing a concentration gradient. As the precursor concentration reaches the saturation state around the interface, seed crystals form along the organic template. Crystal growth is then driven by the concentration gradient at room temperature. The overall crystal growth process is illustrated in Figure S1a and the crystal picture is shown in Figure S1b, highlighting the ease of the synthesis process at room temperature without requiring additional heat and pressure.

Single-crystal X-ray diffraction (SCXRD) measurements were used to obtain the structure models of R/S-DPEDPb_3_Br_8_·H_2_O shown in Figure 1b. The racemic crystal ((Rac-DPED)_2_Pb_1_Br_6_·4H_2_O) exhibits a distinct structure, as shown in Figure S2. Crystallographic data and structure refinement information are summarized in Table S1. The Flack parameters from SCXRD (*R* = 0.003(4); *S* = −0.014(5)) confirm that chirality is maintained during the synthesis. The asymmetric unit contains 12 unique lead atoms (empirical formula DPEDPb_3_Br_8_·H_2_O, *Z* = *Z*′ = 4). The Pb atoms are either 6-coordinate with distorted octahedral geometry (Pb1–Pb4) or 8-coordinate with distorted bicapped trigonal prismatic geometry to give polyhedra with one square face and 10 triangular faces (Pb5–Pb12). Examples of 6-coordinate or 8-coordinate polyhedra with bond lengths are depicted in Figure 1c. The distortion values (Δ*d*) of the Pb–Br bond length are calculated using the equation , where *n* represents the Pb atoms coordination number, *d* denotes the mean Pb–Br bond distance over the entire structure, and *d_n_* signifies the individual Pb–Br bond distances. The Δ*d* values of octahedra with Pb atom of 6 and 8 coordinates are 4.5 × 10^–3^ and 2.0 × 10^–3^, respectively. These Δ*d* values are higher than those in most low-dimensional hybrid crystals, such as 3.26 × 10^–3^@DMABAPbBr_4,_^26^ 1.63 × 10^–3^@PEA_2_PbBr_4_,^27^ and 0.94 × 10^–3^@BA_2_PbBr_4._^28^ This indicates the high degree of distortion in the inorganic framework of DPED-based CHLHs due to the formation of hydrogen and halogen bonding between the bromine and ammonium groups (and water molecules). The corresponding hydrogen and halogen bonding within the structure is listed in Table S2. Such distortion facilitates effective chirality transfer, thereby amplifying its chiroptical properties.

The lead bromide polyhedra pack to form zigzag layers parallel to the (011) plane (Figure 1b). When viewed down the crystallographic *a*-axis, the polyhedra pack as two rows of 6-coordinate edge-sharing polyhedra, as is typical of CHLH layer structures, followed by four rows of 8-coordinate polyhedra. The packing of these 8-coordinate polyhedra causes kinking in the layer to generate the zigzags. The 8-coordinate polyhedra are face-sharing parallel to the *a*-axis, which corresponds to face-sharing of the “trigonal” faces of the bicapped trigonal prisms. The polyhedra are edge-sharing parallel to the (011) plane direction.

Unlike conventional low-dimensional hybrid crystals where distinct inorganic framework layers and organic spacer layers form, the inorganic framework layers in R-DPEDPb_3_Br_8_·H_2_O have very close interactions. The shortest distance between the two inorganic layers is a nonbonding Br···Br distance of only 3.7 Å, shorter than twice the van der Waals radius of Br–3.72 Å. The DPED organic spacers occupy channels formed by the stacking of a zigzag inorganic Pb–Br framework (Figure 1b). The closely spaced Br···Br atoms are stabilized by hydrogen bonding interactions with the ammonium groups of DPED. The close interaction between the inorganic layers reduces the dielectric confinement and quantum confinement effect.^29^ Moreover, larger conjugated organic molecules, compared to aliphatic or smaller aromatic cations, are likely to possess higher dielectric constants, thereby diminishing dielectric confinement effects.^30^ It is known that the reduced confinement effects decrease both the excitonic binding energy and spin relaxation rate in low-dimensional halide perovskites.^31,32^ The PXRD pattern, as shown in Figure 1d, the measured and simulated results, indicating the high purity and crystallinity of synthesized crystals. Thermogravimetric analysis (TGA) showed the onset temperature of weight loss at 242 °C, demonstrating high thermal stability, as shown in Figure S3, making the compounds suitable candidates for practical application.

**Figure 2:**
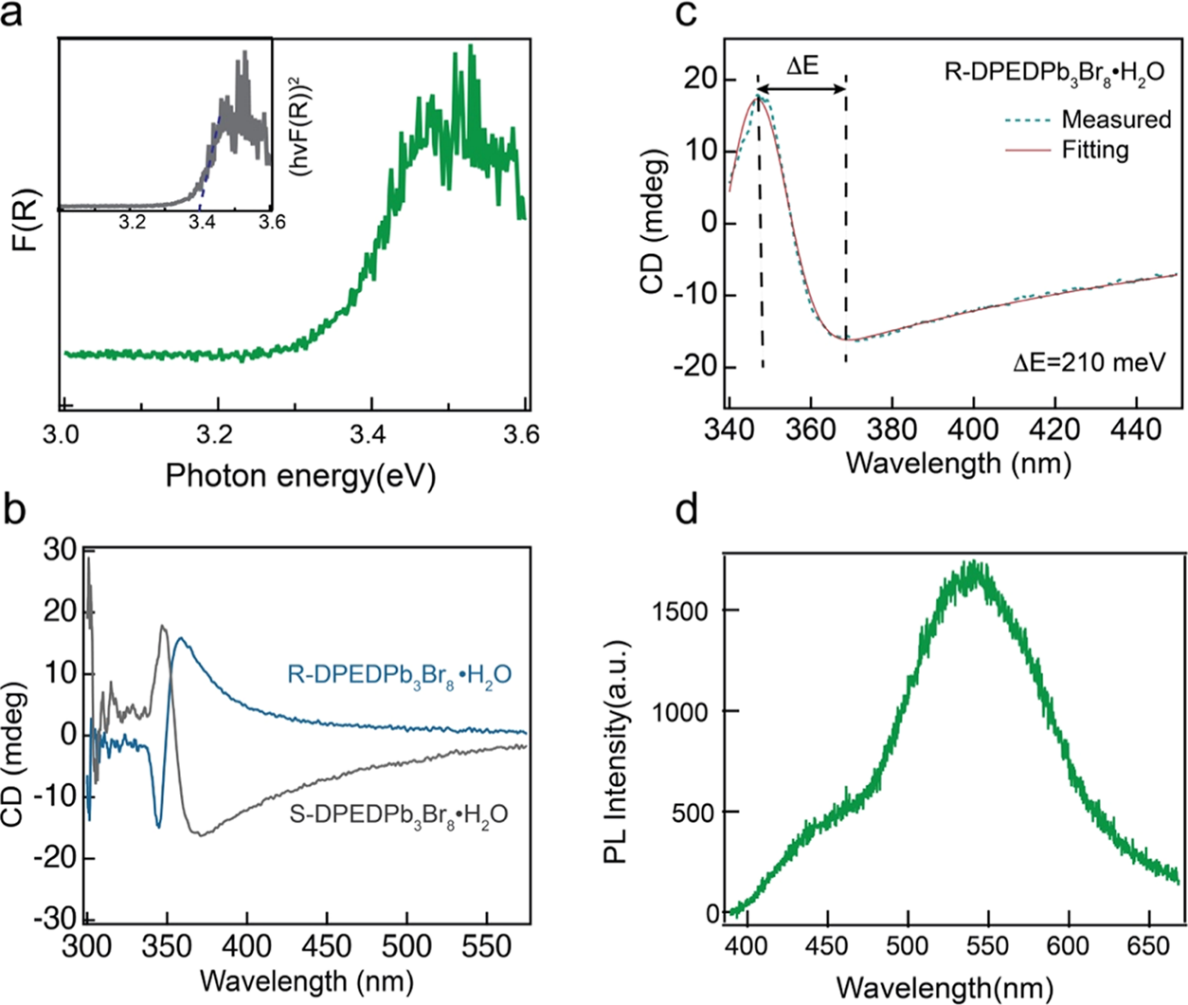
(a) Optical absorption spectra of R-DPEDPb_3_Br_8_·H_2_O obtained from diffuse reflectance measurements and converted using the Kubelka–Munk function. Inset data represent a Tauc plot. (b) CD absorption spectra of R/S-DPEDPb_3_Br_8_·H_2_O thin films. (c) Fitted result was obtained from the CD spectra of S-DPEDPb_3_Br_8_·H_2_O with Δ*E*. (d) Steady-state photoluminescence spectrum of the R-DPEDPb_3_Br_8_·H_2_O single crystal at room temperature.

The optical band gap of the DPED hybrid crystal was determined using diffuse reflectance spectroscopy. The reflectance value (*R*) was converted to *F*(*R*) using the Kubelka–Munk function, , and plotted as a function of photon energy. Figure 2a presents the diffuse reflectance spectra of R-DPEDPb_3_Br_8_·H_2_O, showing absorption primarily in the band gap. The band gap energy, *E*_g_ = 3.4 eV, was calculated using a Tauc plot, which plots (*F*(*R*)*h*ν)^2^ against the photon energy as shown in the inset of Figure 2a. We also measured the UV–vis absorption spectrum of the spin-coated thin film by dissolving single crystals. The spectrum exhibited features identical to those of single crystals measured in the diffuse reflectance (Figure S4). The band gap energy is consistent with the calculated value of ∼3.5 eV. The absence of strong excitonic absorption further substantiates the evidence for low exciton binding energy.^32^ To validate the chirality transfer from chiral organic molecules to inorganic building blocks, circular dichroism (CD) was measured on R-DPEDPb_3_Br_8_·H_2_O thin films, which were prepared by dissolving the as-synthesized single crystal in dimethylformamide (DMF) and deposited onto the glass substrates. The CD spectra showed opposite bisignate results at the peak position of R- and S-DPEDPb_3_Br_8_·H_2_O thin films around 370 nm, which correlates to the absorption edge, as shown in Figure 2b. Excitonic-state splitting (Δ*E*) was calculated by fitting the CD result and elucidating the role of the chiral organic spacer in shaping the chiroptical properties of CHLH.^33^ As shown in Figure 2c, a Δ*E* value of 210 meV was extracted from the fitted CD spectra (the fitting function is described in Supporting Information note 1), which is higher than naphthyl-ethylamine (NEA)-based CHLHs (55.8 and 83.6 meV for two different NEA organic spacers^34^). Both CD and the results attest to a robust and effective chirality transfer process from the organic spacer to the inorganic framework by incorporation of the DPED ligands. The bisignate result originates from the energy-state perturbation by chiral organic molecules.^35^

Steady-state photoluminescence (PL) spectra of the single crystals were measured at room temperature by using a 375 nm continuous-wave (CW) linearly polarized laser. The PL spectra revealed a strong white emission with a broadening peak stemming from the self-trapped emission (Figure 2d), commonly shown in low-dimensional hybrid crystals.^36,37^ The home-built system was designed to measure the CP-PL using a quarter-wave plate (QWP) and a linear analyzer between the sample and the detector. The system was carefully calibrated with a laser and reference samples to negate any artificial influence on the CP-PL signal. Measurement of CP-PL involved converting the circular to linear polarization by using the QWP, followed by measuring the polarization ratio by rotating the linear analyzer. The polarization ratio (*P*) of CP-PL was determined using the equation , where *I*_min_ and *I*_max_ represent the highest and lowest CP-PL integrated intensities, respectively. Figure S5 shows a polarization ratio of approximately 0.06 for R-DPEDPb_3_Br_8_·H_2_O as a function of azimuth angles of the linear analyzer.

**Figure 3:**
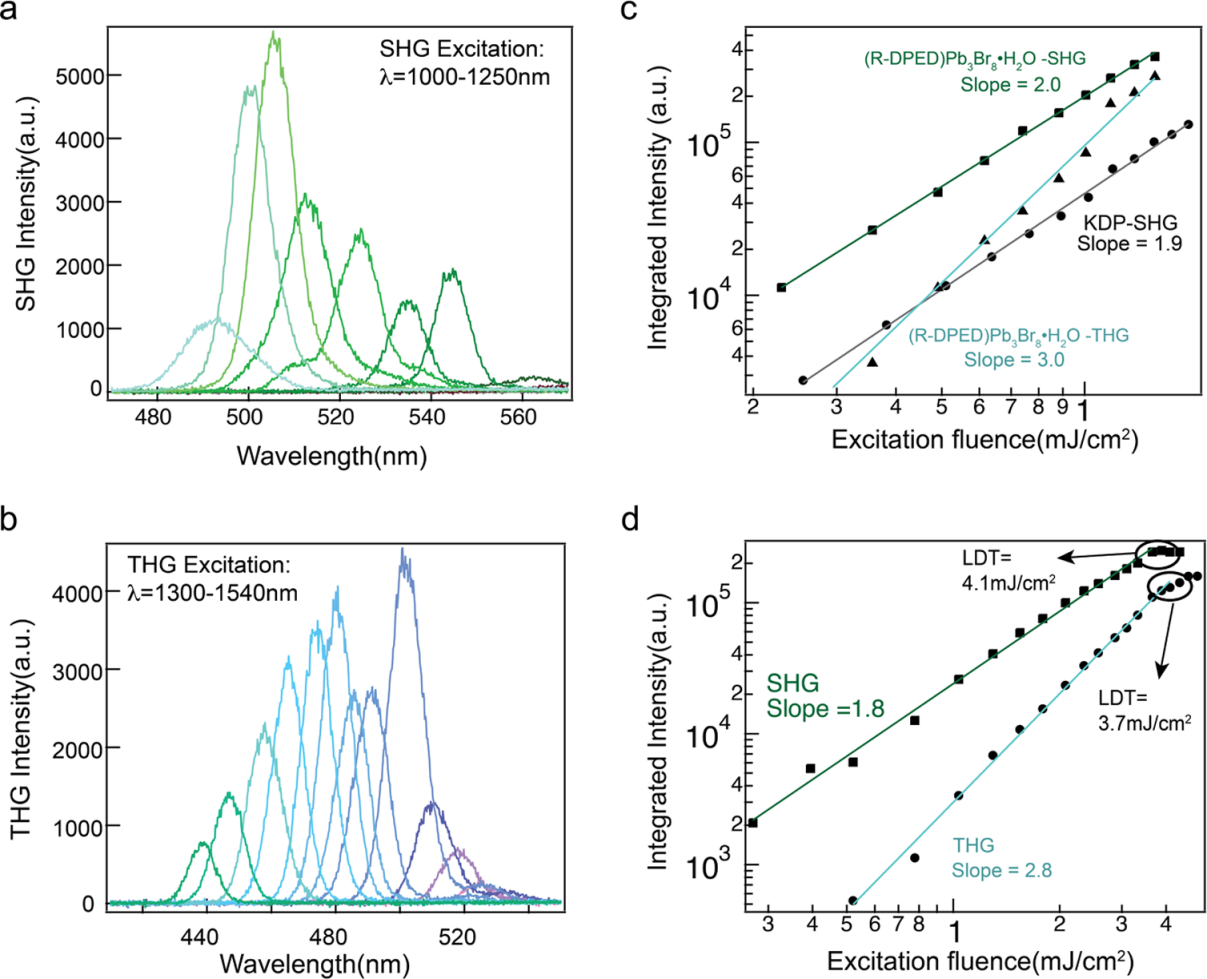
(a) Wavelength-dependent nonlinear optical properties of R-DPEDPb_3_Br_8_·H_2_O at room temperature, SHG with 1000–1250 nm excitation and (b) THG with 1300–1540 nm excitation. (c) Power-dependent SHG, THG, and KDP intensity. (d) Laser damage threshold measurements of SHG and THG properties.

The NLO properties of chiral R-DPEDPb_3_Br_8_·H_2_O, specifically SHG and THG, were investigated using a home-built optical setup. The wavelength-dependent measurements spanned from the excitation wavelength of 1000–1240 and 1300–1560 nm with 20 nm intervals, respectively, as shown in Figure 3a,b. The longer wavelength range corresponds to the SHG signal near the self-trap exciton state and covers the broad emission, while the shorter wavelength zone pertains to the THG signal that fills the state close to the free exciton state. To verify the SHG and THG signatures, the excitation power-dependent SHG and THG was performed using 1064 and 1300 nm femtosecond lasers. The SHG results are presented on the log–log scale in Figure 3c, with the result of KDP crystal as a comparison. The SHG showed a slope of 2, which fits the second order power law, and the SHG intensity of R-DPEDPb_3_Br_8_·H_2_O is 10-fold greater than KDP crystal under the same condition, indicating the high SHG performance of R-DPEDPb_3_Br_8_·H_2_O. For THG, a slope of 3.0 was obtained when fitted to the THG power law. The angle-resolving polarization-dependent SHG results were measured with the linear polarizer and a half-wave plate (HWP). The polarization of the input linearly polarized laser was fixed, and the sample was rotated during the measurement. The angle was defined as the value of the crystal axis away from its origin direction (laboratory coordinates). Figure S6 shows the SHG intensity as a function of the azimuth angle of the HWP with different crystal orientations, exhibiting 2-fold symmetry in all angles, which is consistent with the C1 point group and demonstrates the lack of inversion symmetry in R-DPEDPb_3_Br_8_·H_2_O. Furthermore, the laser damage threshold (LDT) was identified under high-power fluence for SHG and THG. The SHG and THG signals kept the quadratic relationship up to 4.1 and 3.7 mJ/cm^2^ power fluence, respectively. At higher power fluence, a constant value was maintained, as shown in Figure 3d. These power fluences correspond to the laser damage threshold, indicating the stability of R-DPEDPb_3_Br_8_·H_2_O under high laser power fluence conditions.

R-DPEDPb_3_Br_8_·H_2_O generates CP-PL in accordance with the circularly polarized emission rule. Under the linearly polarized light excitation with left-handed or right-handed chiral particles, spins can be excited from the ground state (|*G*⟩) to the exciton state (|*J*_*Z*_⟩_*X*_) with angular momentum projection along the *z*-axis, *J*_*Z*_ = ± 1. As an example, we take the right-handed chiral crystal, which carries +1 angular momentum along the *z*-axis. Excitons with one type of spin can be promoted from |*G*⟩ to |+1⟩_*X*_, whereas another type of spin transition is forbidden by the selection rule, which creates spin polarization. As spin flipping occurs, spins in the |+1⟩_*X*_ state flip to |−1⟩_*X*_, diminishing the spin polarization in the excited state. Consequently, it is crucial to measure the spin relaxation rate. CP-PL occurs when the radiative recombination occurs between the electrons with spin polarization and holes in the ground state, and slow spin relaxation rates are important for CP-PL performance.

**Figure 4:**
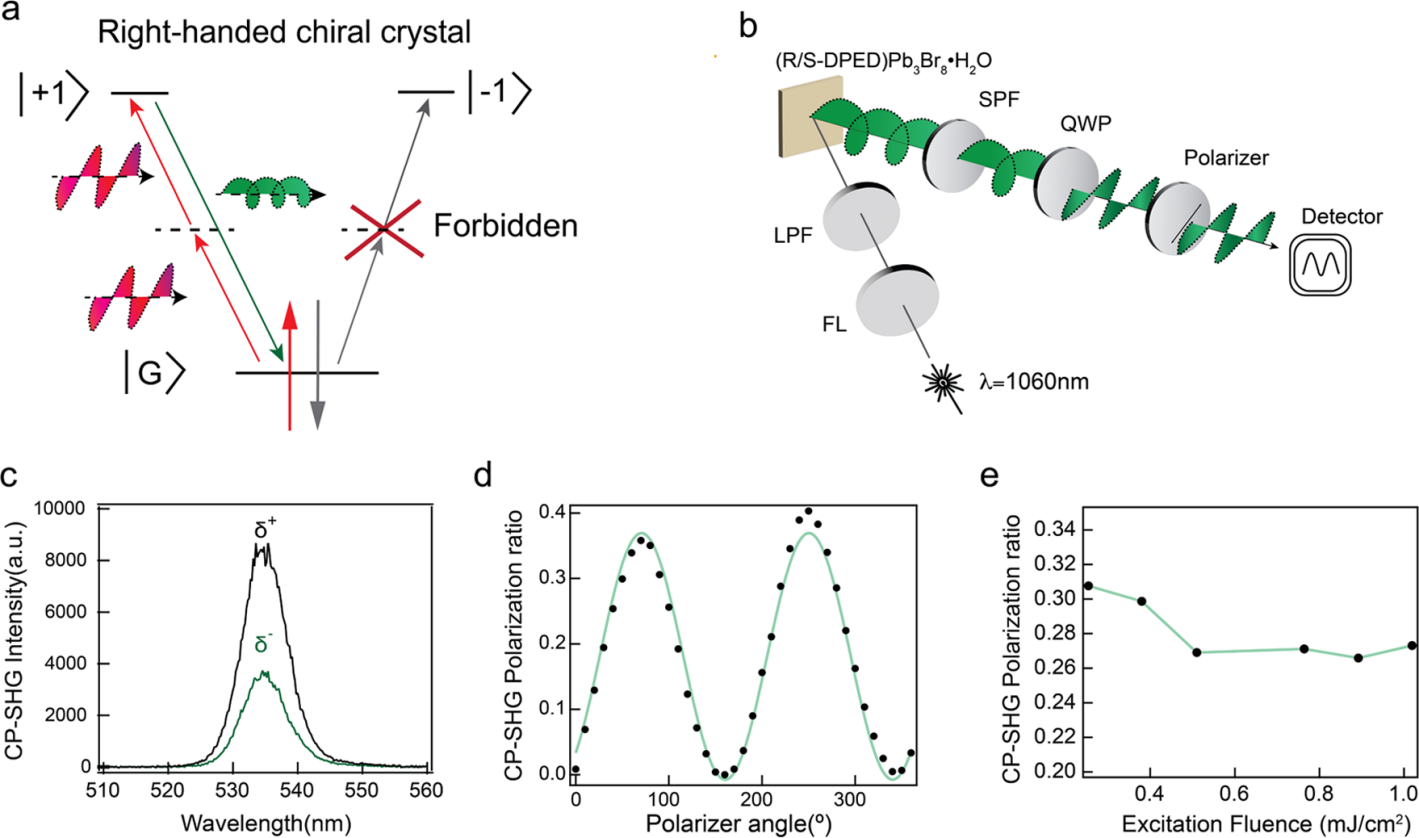
(a) Schematic diagram of the spin selection rule in illustrating CP-SHG. (b) Diagram of the CP-SHG measurement setup. SHG spectrum detected with right/left-handed polarization (c) and polarization ratio plot as a function of the polarizer angle (d) of CP-SHG from R-DPEDPb_3_Br_8_. (e) Measured CP-SHG polarization ratio as a function of excitation pump power fluence of the infrared laser.

We propose a spin selection rule for CP-SHG in accordance with the observed CP-PL. Initially, electrons are excited by two photons through the virtual intermediate state and create the exciton with spin polarization in the excited state. Subsequently, the radiative recombination happens between spin-polarized electrons and holes and circularly polarized light is emitted. The excitation and emission processes are depicted in Figure 4a. Similar to CP-PL, the spin relaxation rate potentially impacts the CP-SHG dynamics. CP-SHG plays an essential role in generating high-energy circularly polarized light, especially in challenging wavelengths, such as the ultraviolet range. R-DPEDPb_3_Br_8_·H_2_O offer an innovative way to directly generate the CP-SHG. Figure 4b shows the CP-SHG measurement setup, with detailed explanations in the Experimental Procedure section. As a calibration experiment, the *P* (polarization ratio) value of the laser as a function of azimuth angles of the linear analyzer shows a constant intensity (laser fluctuation < 4% in intensity), indicating the stability of the system and no interference of the laser on the CP-SHG signal, as seen in Figure S7. We have shown CP-SHG for both R-DPEDPb_3_Br_8_·H_2_O (Figure 4c) and S-DPEDPb_3_Br_8_·H_2_O (Figure S8). All CP-SHG signals peaked at 533 nm, with noticeable intensity variances observed between right-handed (δ^+^) and left-handed (δ^–^) for R/S-DPEDPb_3_Br_8_·H_2_O.

In Figure 4d, the maximum CP-SHG polarization ratio for R-DPEDPb_3_Br_8_·H_2_O was 0.37, which is higher than most circularly polarized emission (CP-PL, CP-SHG, CP-Up conversion), when compared to other CHLHs, such as 0.03@MAPbBr_3_(CP-PL).^38^ The CP-SHG of another five different crystal batches has been quantified. The observed polarization ratio fell within the narrow range between 0.3 and 0.4, which is shown in Figure S9, demonstrating the consistency and reproducibility of the CP-SHG measurement. The polarization ratio of CP-SHG for S-DPEDPb_3_Br_8_·H_2_O reaches up to 0.25 and has 90° peak-shifting attributed to the opposite chirality of its organic spacer, as shown in Figure S10. Further, we measured SHG from a polycrystalline thin film, as shown in Figure S11. We obtained the SHG signal using high laser power fluence and longer accumulation time due to the significantly weaker signal to compare with the single crystalline sample. To investigate the relationship between spin relaxation and the polarization ratio of CP-SHG, we conducted temperature-dependent and power-dependent CP-SHG measurements. The excitation power-dependent CP-SHG results in Figure 4e displayed two stages of the polarization ratio: an initial decrease in the ratio as power fluence increased, followed by a stabilized ratio despite further increases in power, suggesting that the polarization ratio is governed by two distinct mechanisms, both related to the spin relaxation mechanism. The temperature-dependent CP-SHG was measured between 25 and 50 °C in 5 °C increments as shown in Figure S12. We did not observe a clear trend from the temperature-dependent CP-SHG measurement.

Three potential spin relaxation mechanisms were considered in the above DPED-based CHLHs: Elliott–Yafet (EY),^39^ D’yakonov–Perel’ (DP),^40^ and Bir–Aronov–Pikus (BAP).^41^ The EY mechanism is attributed to phonons and impurity scattering and typically governs spin depolarization in 3D halide perovskites, especially those based on lead-iodide-based perovskites, manifesting temperature-driven effects.^42,43^ The absence of a temperature trend in the CP-SHG measurements (Figure S12) implies that the EY mechanism might not be operative in zigzag-structured CHLHs. However, at the room-temperature regime, the EY mechanism can still influence the spin relaxation rate. The DP mechanisms are the dominant mechanism in noncentrosymmetric systems and semiconductors with elements from groups III–V,^44^ while the BAP mechanism relies on the exciton exchange.^45^ At elevated temperatures (higher than 250 K), both mechanisms exhibit a less temperature-dependent spin relaxation rate compared to the scattering effect.^43,46^ The DP mechanism triggers the spin relaxation through the pseudomagnetic fields created by SOC and the symmetry broken in chiral hybrid lead halides.^47^ The exciton binding energy plays a critical role in the spin relaxation rate () and is power-independent.^29^ In the high-power fluence zone, the constant polarization ratio can contribute to the DP spin relaxation mechanism. The zigzag layer structure with reduced dielectric confinement and quantum confinement and the lack of an excitonic absorption peak in the UV–vis spectrum indicate relatively low exciton binding energy, leading to a high polarization ratio at a high excitation power fluence regime.

**Figure 5:**
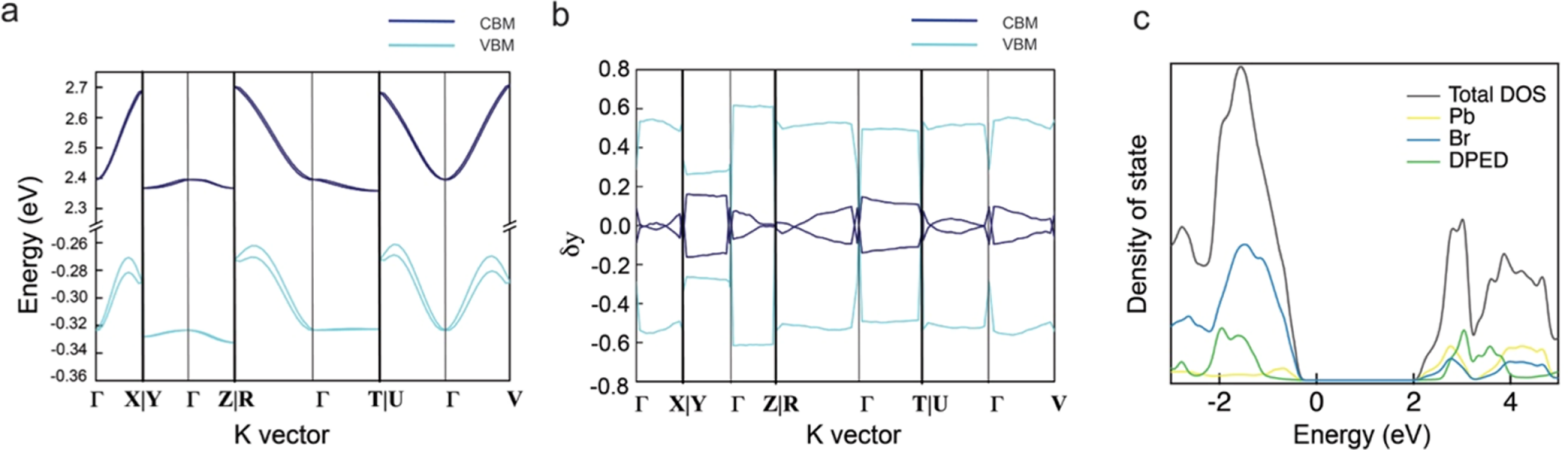
(a) Electronic structure, (b) momentum-dependent spin polarization along the *y* direction, and (c) total and partial DOS for R-DPEDPb_3_Br_8_·H_2_O.

The band structure and momentum-dependent spin polarization were calculated by first-principle DFT calculations. The close interaction of the inorganic layers in R-DPEDPb_3_Br_8_·H_2_O to form 1D cationic channels, rather than the distinct inorganic and organic layers, as is often observed in conventional 2D CHLHs, affects the electronic structure. This was validated by the DFT calculations of R-DPEDPb_3_Br_8_·H_2_O depicted in Figure 5a. As illustrated in the crystal structure, the Pb–Br motif features unique 8-coordinated Pb–Br building blocks, causing the indirect band gap nature of the electronic structure.^48^ The bands are dispersive along ΓX, ΓR, ΓU, and ΓV high-symmetry directions, all of which are within the plane of the inorganic framework and approximately parallel to the octahedral connections, as shown in Figure S13. The remaining *K* vector directions that are parallel to the inorganic plane–organic spacer stacking direction displayed flat bands due to reduced electronic dimensionality by the organic spacer. Also, the distinctive zigzag layer structure induces two types of Pb–Br unit connections—edge-sharing and face-sharing. These connections contribute to band flattening at both the valence band minimum (VBM) and the conduction band maximum (CBM), attributed to the reduced band dispersion associated with the edge-sharing and face-sharing configurations.^49^

Spin polarizations in VBM and CBM were calculated by momentum-dependent spin polarization, as shown in Figures 5b and S14. The top two dispersive valence bands show opposite spin polarizations with the polarization direction mainly along y from momentum-dependent spin polarization, indicating spin-splitting that is attributed to the SOC and lack of inversion symmetry. In the DP-governed low-power fluence zone, the spin relaxation was influenced by the strong SOC and low exciton binding energy, contributing to the high polarization ratio of CP-SHG. The conduction bands, mainly composed of Pb 6p orbitals as deduced from the density of states calculations, are shown in Figure 5c and exhibit notable differences in R-DPEDPb_3_Br_8_·H_2_O compared with traditional 2D CHLHs. The 8-coordinated Pb configuration in R-DPEDPb_3_Br_8_·H_2_O leads to significant distortion of the lead p orbitals, diminishing their interchange with the electronic state and reducing the symmetry of p orbital degeneracy.^50^ Together, these alterations impact SOC at the CBM, substantially reducing spin-splitting and, consequently, slow down the spin relaxation rate.^51^ In the regions of the low excitation fluence regime, CP-SHG is primarily governed by the BAP mechanism. Here, the exciton density is the dominant factor, indicating the influence of power fluence.^45^ As the power is increased, leading to an increase in exciton density, the spin relaxation rate also increases, consequently lowering the CP-SHG polarization ratio. This trend aligns with the observed results in areas of low-power fluence steered by the BAP mechanism.

## Conclusions

In summary, R/S-DPEDPb_3_Br_8_·H_2_O was synthesized via the oil–water interface method, adeptly bypassing challenges such as steric effects and a large cation size. The inorganic framework distortion was induced by the formation of 8-coordinate Pb polyhedra and strong hydrogen and halogen bonding, which increased the thermal stability and chiroptical properties. The very close contact between the inorganic layer led to a decrease in both dielectric and quantum confinement effects, reducing the excitonic binding energy. R/S-DPEDPb_3_Br_8_·H_2_O manifested significant nonlinear optical SHG and THG and also exhibited high LDT, which suggested that R/S-DPEDPb_3_Br_8_·H_2_O holds promising potential for applications in various optoelectronic devices. A standout feature of R/S-DPEDPb_3_Br_8_·H_2_O is its ability to control the polarization of SHG, achieving a polarization ratio that surpasses those of most other CHLH materials (as high as 0.37 for the R type and 0.25 for the S type). Our investigations into spin relaxation mechanisms, based on temperature and power-dependent CP-SHG, have revealed that both BAP (in low power fluence regions) and DP (in high-power fluence regions) mechanisms predominantly govern spin relaxation. The theoretical computations of R-DPEDPb_3_Br_8_·H_2_O exhibited strong spin-splitting and high spin polarization, which contribute to the high polarization ratio observed in R-DPEDPb_3_Br_8_·H_2_O. Our study has opened new horizons in advancing the spin polarization for chiroptical materials.

## Experimental Procedure

### Crystal Synthesis

#### Growth of 2D R/S-DPEDPb_3_Br_8_·H_2_O and Rac-(DPED)_2_Pb_1_Br_6_·4H_2_O Single Crystals

To synthesize R/S-DPEDPb_3_Br_8_·H_2_O single crystals, we used a two-step water–oil interface method. First, 0.12 mmol of PbBr_2_ was dissolved in a 4 mL solution containing 1 mL of 48% HBr and 3 mL of DI water, at room temperature, and stirred for 45 min. As a second step, 0.4 mmol of the DPED ligand was added to the PbBr_2_ solution and stirred for 20 min. The solution was filtered by using a 0.2 μm filter. A total of 800 μL of silicone oil was added to the filtered solution to form the water–oil interface. The solution was left undisturbed for 1 week, during which time transparent crystals formed at the water–oil interface. The synthesis of Rac-(DPED)_2_Pb_1_Br_6_·4H_2_O employed the same procedure as for R/S-DPEDPb_3_Br_8_·H_2_O, with the modification of using mixed 0.2 mmol of S-DPED and 0.2 mmol of R-DPED.

### Characterization

#### UV–Vis Absorption Measurements

Diffused reflectance and UV–vis absorption measurements were performed using a UV–vis–NIR spectroscopy from Hitachi U4100.

#### Thermal Analysis Measurements

Thermogravimetric analysis measurement is conducted by a TGA 5500 from TA Instruments.

#### Single-Crystal XRD Measurements

The crystals were centered on the goniometer of a Rigaku Oxford Diffraction Synergy-S diffractometer equipped with a HyPix6000HE detector and operating with Mo Kα radiation. The data collection routine, unit cell refinement, and data processing were carried out with the program CrysAlisPro.^52^ The Laue symmetries were consistent with triclinic space groups *P*1 and *P*1̅. As the organic cation in the compounds is known to be enantiomerically pure, the noncentrosymmetric space group *P*1 was chosen. The structure of the R-enantiomer was solved using SHELXT^53^ and refined using SHELXL^54^ via Olex2.^55^ The coordinates for the starting model of the S-enantiomer were obtained by using inverted coordinates from the final model of R-enantiomer. This method kept atom labeling consistent between the two models for ease of comparison. The final refinement models involved anisotropic displacement parameters for nonhydrogen atoms and a riding model for all hydrogen atoms. For the water molecules, AFIX was used to generate an idealized geometry. Then, the water molecules were inspected for their chemical viability. For all four water molecules in the asymmetric units, the two lone pairs on the oxygen are clearly H-bond acceptors with ammonium groups (N–H···O). To achieve chemical viability, the molecules should achieve approximate tetrahedral geometry around the oxygen (2O–H bonds + 2O···H interactions). When the geometry deviated substantially from the tetrahedral form, DFIX restraints with nearby atoms were used to “rotate” the water into approximate tetrahedral geometry. In the final model, all four water molecules formed one O–H···Br interaction and one potential O–H···π (aromatic) interaction. The absolute configurations were established from anomalous dispersion effects [R-enantiomer: Flack *x* = −0.005(4); Parson *q* = 0.003(4);^56^ Hooft *y* = −0.023(4);^55,57^ S-enantiomer: Flack *x* = −0.014(5); Parson *q* = −0.014(5); Hooft *y* = −0.042(5)].

#### Circularly Polarized Photoluminescence Measurements

The steady-state PL was measured using a 375 nm continuous-wave laser, and the signal was collected by a nitrogen-cooled charge-coupled device (CCD) camera equipped with a monochromator. The CP-PL measurement used the same laser, and the signal of CP-PL passed through a quarter-wave plate and a linear polarizer before being collected by a CCD camera. The intensity of the PL as a function of the azimuth angle of the linear polarizer is shown in Figure S5.

#### SHG and THG Measurements

We used a femtosecond laser generated from the Astrella-F-1K one-box femtosecond amplifier (Coherent) with an optical parametric amplifier system. The laser light passed through the neutral density filter and long pass filter before focusing on the sample through a focus lens. A short pass filter was placed between the sample and the detector to rule out the influence of the laser. The final signal was collected with a CCD camera. The power was adjusted by changing the neutral density filter for power-dependent SHG and THG measurements. Measurements of wavelength-dependent SHG and THG were conducted at a constant excitation laser power fluence of 0.25 mJ/cm^2^ (1 kHz, 1000 μm in diameter spot femtosecond laser). For polarization-dependent SHG and THG, before the laser focuses on the sample, it passes through a linear polarizer and a half-wave plate. The linear polarization of the SHG was tested by rotating the half-wave plate.

#### Circularly Polarized SHG Measurements

CP-SHG measurements were conducted by modifying the SHG measurement setup. A quarter-wave plate and a linear polarizer were placed between the sample and the detector, as shown in Figure 4b. Initially, the SHG signal passes through the quarter-wave plate for polarization reduction, which converts the circularly/elliptically polarized light to the linear polarized light and is followed by the linear polarizer to detect the polarization. The orientation of the quarter-wave plate remained constant throughout the measurement, while the rotation of linear polarization was recorded to examine circularly polarized light. The unpolarized light passing through the quarter-wave plate remains unpolarized, whereas linearly polarized light is converted to circularly polarized light. After passing through the quarter-wave plate, both unpolarized and linear polarized light maintain their intensity during rotation of the linear polarizer. In the case of circularly polarized or elliptically polarized light, changes in intensity are observed as a function of the azimuth angle of the linear polarizer. The entire system was calibrated meticulously prior to testing to rule out any artificial signal that could affect the CP-SHG result. The degree of polarization (*P*) of the system and CP-SHG were measured by rotating the linear analyzer. The polarization ratio as a function of the azimuth angle of the linear polarizer is depicted in Figure 4d, indicating the circularly polarized light emitted from the DPED-based crystal.

#### First-Principles DFT and Spin Polarization Calculations

First-principles DFT calculations were performed using the VASP code with projector augmented-wave potentials.^58−60^ A plane-wave energy cutoff of 500 eV and a Γ centered 2 × 1 × 1 *k*-mesh were used. The exchange-correlation interactions were treated with the generalized gradient approximation of the Perdew–Burke–Ernzerhof (PBE) parametrization.^61^ Grimme’s D3 correction was also included to deal with the van der Waals interactions.^62^ Spin–orbit interaction was included in the band structure calculations. Spin polarization along high symmetry lines was calculated using PyProcar.

## Supporting Information Available

Schematic of the oil–water interface crystallization process (Figures S1–S14 and Tables S1–S2); TGA of the R-DPEDPb_3_Br_8_·H_2_O single crystal; ratio and measurement setup of CP-PL of the R-DPEDPb_3_Br_8_·H_2_O single crystal; polarization-dependent SHG and the measurement setup based on different crystal orientations; calibration measurement: laser intensity as a function of the azimuth angle of the linear polarizer and the measurement setup; CP-SHG of the S-DPEDPb_3_Br_8_·H_2_O as a function of the azimuth angle of the linear polarizer; temperature-dependent polarization ratio from CP-SHG of R-DPEDPb_3_Br_8_·H_2_O *K* vector diagram in the crystal structure viewing down the *a*-, *b*-, and *c*-axes; and momentum-dependent spin polarization (PDF)
