# Supplementary material for: Direct Observation of Circularly Polarized Nonlinear Optical Activities in Chiral Hybrid Lead Halides
Source: J Am Chem Soc. 2024 Apr 3;146(17):11835–44. doi: 10.1021/jacs.4c00619 (PMC11066869; doi:10.1021/jacs.4c00619)
Supplement: Supplementary file 1 — ja4c00619_si_001.pdf [file ja4c00619_si_001.pdf]

Supplementary information for

Direct Observation of Circularly Polarized Non-linear Optical Activities in Chiral Hybrid Lead Halides

Sunhao Liu[1], Xiaoming Wang[3], Yixuan Dou[1], Qian Wang[1], Jiyoon Kim[1], Carla Slebodnick[1], Yanfa Yan[3], Lina Quan[1,2]

1. Department of Chemistry, Virginia Tech, Blacksburg, VA 24061, USA
2. Department of Materials and Science Engineering, Virginia Tech, Blacksburg, VA 24061, USA
3. Department of Physics and Astronomy and Wright Center for Photovoltaics Innovation and Commercialization, The University of Toledo, Toledo, OH 43606, USA

Table S1. Crystal and Refinement Data for (S/R-DPED)Pb_3Br_8 •H_2O and racemic crystal.

Identification code	(S-DPED)Pb_3Br_8•H_2O	(R-DPED)Pb_3Br_8•H_2O	(R/S-DPED)$_2Pb_1Br_6$•4H_2O
Empirical formula	$C_{14}H_{20}Br_8N_2OPb_3$	$C_{14}H_{20}Br_8N_2OPb_3$	$C_{28}H_{44}Br_6N_4O_4Pb$
Formula weight	1493.17	1493.17	288.00
Temperature/K	100.00(10)	99.9(2)	99.9(4)
Crystal system	triclinic	triclinic	triclinic
Space group	P1	P1	P-1
a/Å	8.51609(16)	8.51730(10)	7.6541(2)
b/Å	18.1453(3)	18.1496(2)	8.9721(2)
c/Å	19.1684(4)	19.1709(2)	14.5944(4)
α/°	69.4116(18)	69.4380(10)	100.750(2)
β/°	84.8696(15)	84.8970(10)	90.327(2)
γ/°	84.9895(15)	85.0900(10)	106.617(2)
Volume/Å^3	2756.78(10)	2759.05(6)	941.68(4)
Z	4	4	4
ρ_{calc}g/cm^3	3.598	3.595	2.031
μ/mm^{-1}	29.882	29.857	10.891
F(000)	2608.0	2608.0	564
Crystal size/mm^3	0.053 × 0.033 × 0.015	0.11 × 0.07 × 0.02	0.16 × 0.07 × 0.04
Radiation	Mo Kα (λ = 0.71073)	Mo Kα (λ = 0.71073)	Mo Kα (λ = 0.71073)
Index ranges	-12 ≤ h ≤ 12, -25 ≤ k ≤ 25, -27 ≤ l ≤ 27	-14 ≤ h ≤ 14, -31 ≤ k ≤ 30, -32 ≤ l ≤ 32	-12 ≤ h ≤ 13, -15 ≤ k ≤ 15, -25 ≤ l ≤ 24
Reflections collected	91743	114122	26956
Independent reflections	33301 [R_{int} = 0.0486, R_{sigma} = 0.0646]	51415 [R_{int} = 0.0421, R_{sigma} = 0.0693]	9088 [R_{int} = 0.0530, R_{sigma} = 0.0581]
Data/restraints/parameters	33301/481/1029	51415/3/1029	9088/12/226
Goodness-of-fit on F^2	0.976	0.942	1.000
Final R indexes [I>=2σ (I)]	R_1 = 0.0404, wR_2 = 0.0732	R_1 = 0.0381, wR_2 = 0.0577	R_1 = 0.0417, wR_2 = 0.0924
Largest diff. peak/hole / e Å^{-3}	2.00/-1.29	2.83/-2.23	4.74/-3.82
Flack parameter	-0.014(5)	0.003(4)	N/A

Table S2. Details of hydrogen and halogen bonds of R-(DPED)Pb_3Br_8 •H_2O.

**Hydrogen bonding**	**Distance D-A**	**Angle D-H...A**	**Hydrogen bonding**	**Distance D-A**	**Angle D-H...A**
N2 -H…Br16	3.36	157.11	N3 -H…Br28	3.27	156.35
N1 -H…Br10	3.73	148.02	N3 -H…Br19	3.29	173.93
N1 -H…Br16	3.36	134.92	N4 -H…Br27	3.31	173.75
N7 -H…Br25	3.29	174.18	N4 -H…Br27	3.3	175.52
N2 -H…Br30	3.37	164.26	N2 -H…O1 (water)	3.09	149.57
N1 -H…Br10	3.73	148.02	N1 -H…O1 (water)	2.79	172.59
N1 -H…Br29	3.51	137.79	N5 -H…O3 (water)	3.45	129.72
N5 -H…Br17	3.3	152.01	N6 -H…O3 (water)	2.72	161.3
N5 -H…Br32	3.68	124.94	N7 -H…O4 (water)	2.89	142
N5 -H…Br17	3.3	152.01	N8 -H…O4 (water)	2.88	170.96
N5 -H…Br32	3.68	124.94	N3 -H…O2 (water)	2.91	148.94
N5 -H…Br15	3.4	152.64	N4 -H…O2 (water)	2.85	171.81
N5 -H…Br29	3.47	148.98	O4 (water)H…Br24	3.28	158.62
N6 -H…Br18	3.33	153.3	O2 (water)H…Br20	3.26	159.79
N6 -H…Br32	3.38	158.53	O3 (water)H…Br1	3.34	140.97
N7 -H…Br14	3.25	148.62	O3 (water)H…Br29	3.52	136.68
N7 -H…Br21	3.68	125.84	**Halogen bonding**	**Distance D-A**	**Angle D-N...A**
N7 -H…Br25	3.29	174.18	C-N4…Br31	3.377	170.15
N8 -H…Br11	3.31	176.26	C-N7…Br23	3.385	177.04
N8 -H…Br21	3.32	169.58	C-N8…Br13	3.277	171.88

Supplementary information Note 1

The data fitting was performed using a bimodal Gaussian function to model the relationship between the independent variable X and the dependent variable Y. The bimodal Gaussian function used is given by:

$$f(x) = a_1 \exp\left(-\left(\frac{x - b_1}{c_1}\right)^2\right) + a_2 \exp\left(-\left(\frac{x - b_2}{c_2}\right)^2\right) + \text{base}$$

where $a_{,} b_{,} c_{,} a_{,} b_{,}$, and base are the parameters to be optimized during the fitting process. The fitting was implemented in R programming language. The initial parameter values were set to $a_1 = 50,\ b_1 = 350, c_1 = 15, a_2 = -60, b_2 = 100, c_2 = 280$, and base = 10. The fminsearch function from the R optimx package was used to minimize the sum of squared residuals between the observed data and the bimodal Gaussian function. The optimal parameter values were determined through an iterative process by adjusting the parameter values to minimize the sum of squared residuals.

$$f(x) = 38.06081464 * \exp\left(-\left(\frac{x-346.47712517}{10.77124154}\right)^2\right) - 855.97944205 * \exp\left(-\left(\frac{x-419.91565276}{397.15153989}\right)^2\right) - 0.08611532$$

The goodness of fit was evaluated using the coefficient of determination (R-squared = 0.9937144), which quantifies the proportion of variance in the dependent variable that is explained by the bimodal Gaussian function. The fitted curve was then plotted against the original data for visual inspection.

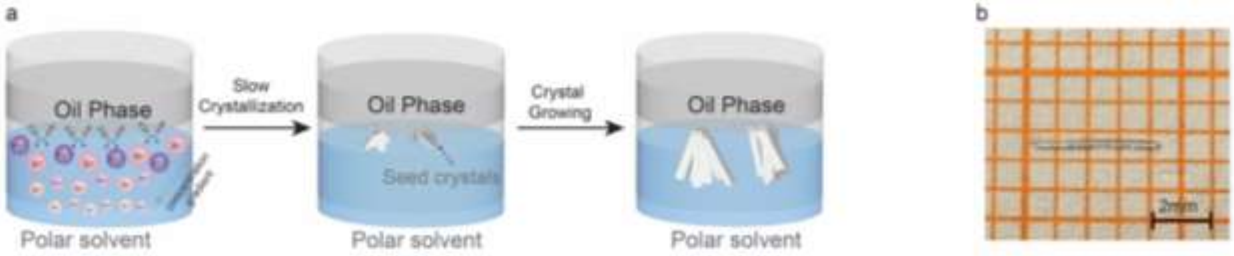

Figure S1. (a) Schematic of oil-water interface crystallization process. (b) crystal photo of R-(DPED)Pb_3Br_8•H_2O.

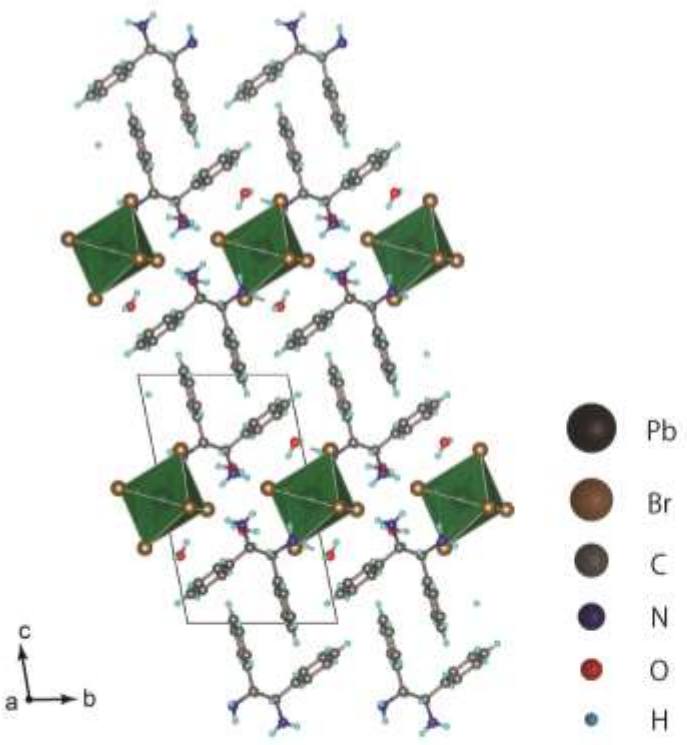

Figure S2. Crystal structure of racemic crystal (R/S-DPED)$_2$Pb$_1$Br$_6$•4H_2O.

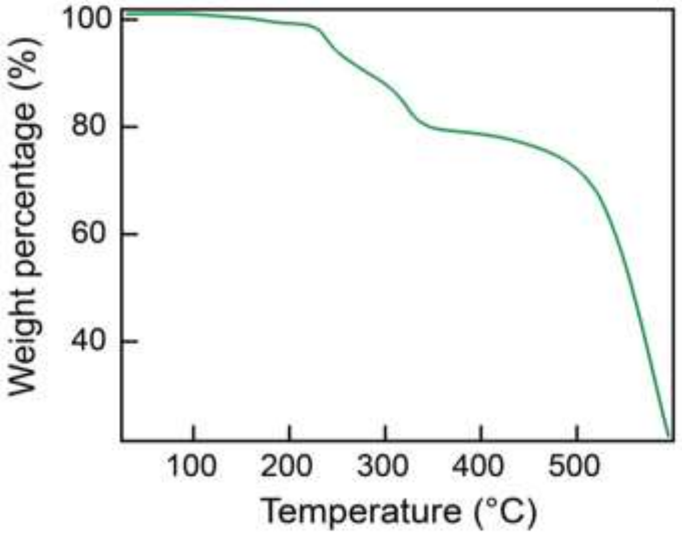

Figure S3. TGA of R-(DPED)Pb_3Br_8•H_2O single crystal.

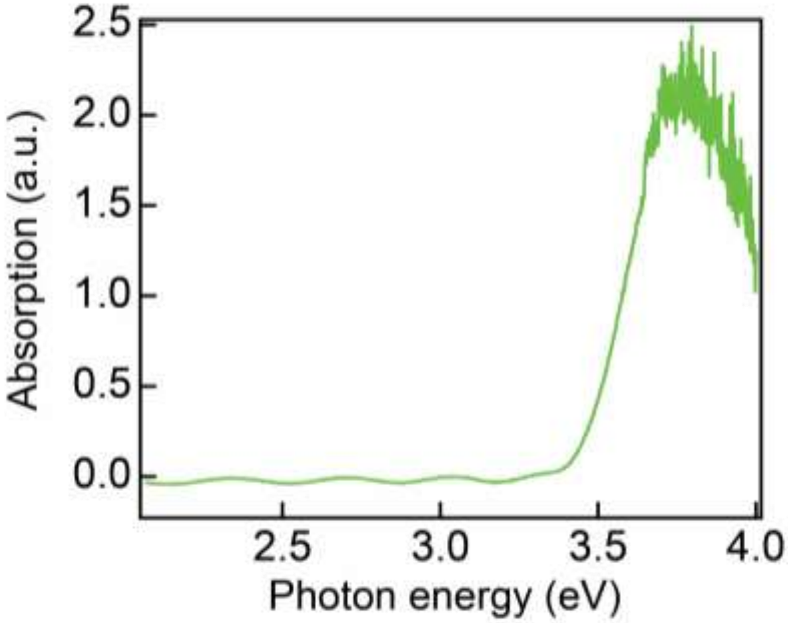

Figure S4. UV-vis absorption spectrum of thin film of R-(DPED)Pb_3Br_8•H_2O.

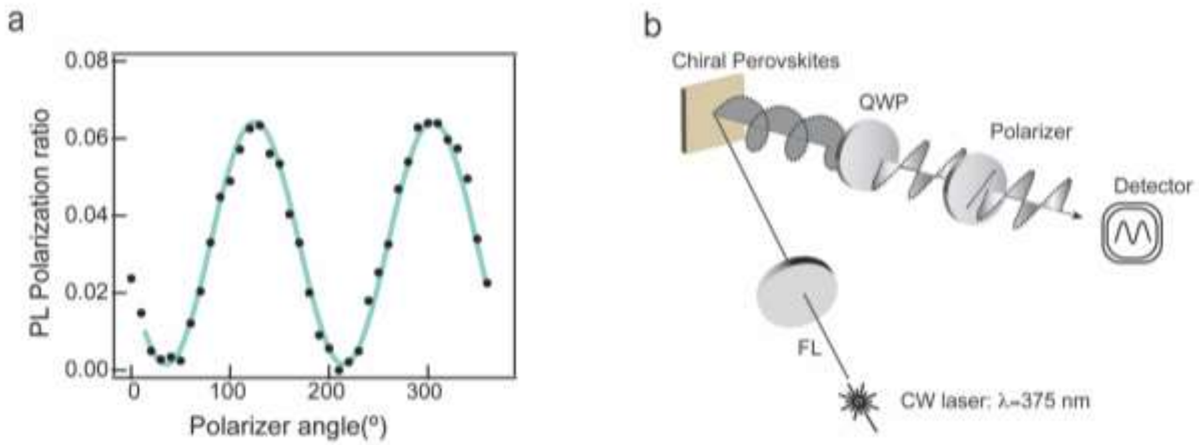

Figure S5. (a) CP-PL ratio of R-(DPED)Pb_3Br_8•H_2O single crystal. (b) Illustration of the CP-PL measurement set up.

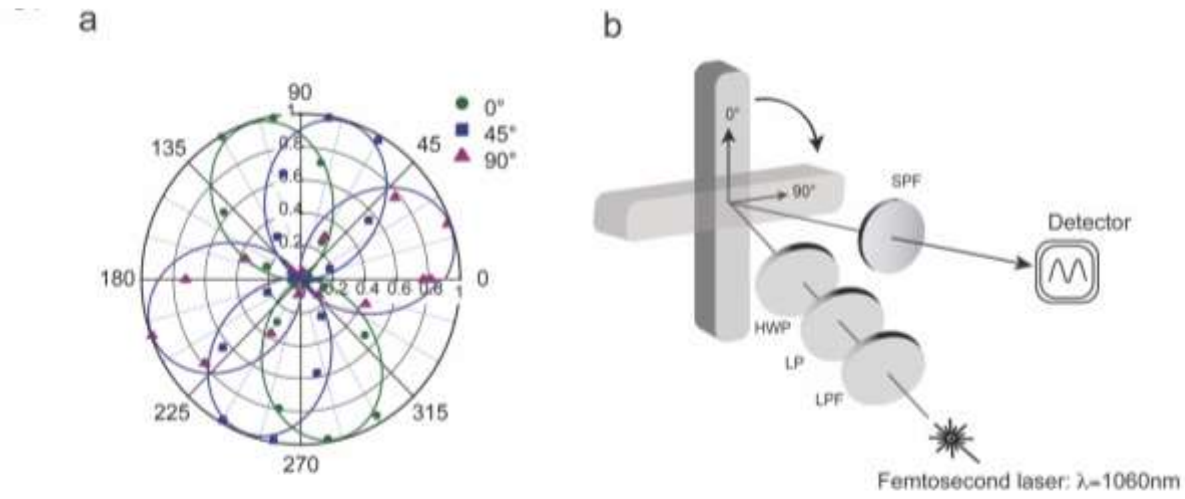

Figure S6. (a) Polarization-dependent SHG from R-(DPED)Pb_3Br_8•H_2O and (b) the measurement setup based on different crystal orientations. (LPF - long pass filter, LP - linear polarizer, HWP - half-wave plate, SPF - short pass filter)

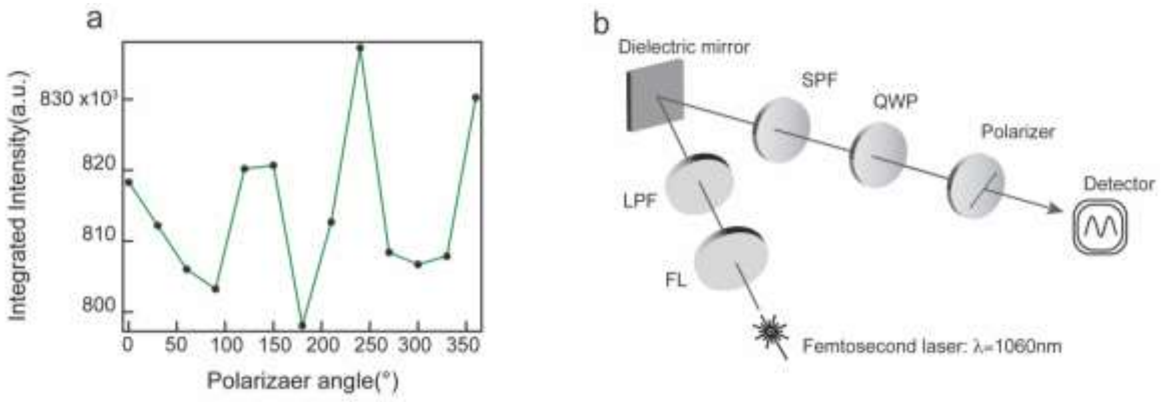

Figure S7. Calibration measurement: laser intensity as the function of azimuths angle of the linear polarizer (a) and the measurement setup (b).

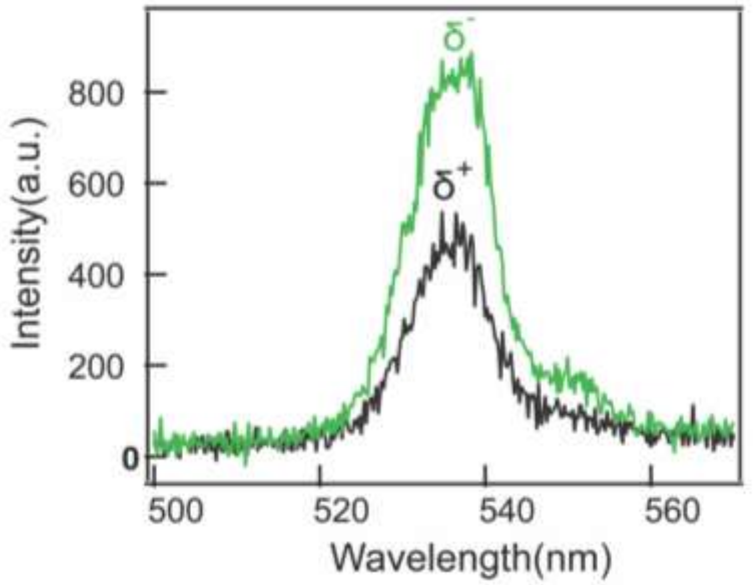

Figure S8. CP-SHG signal from the S-(DPED)Pb_3Br_8•H_2O single crystal.

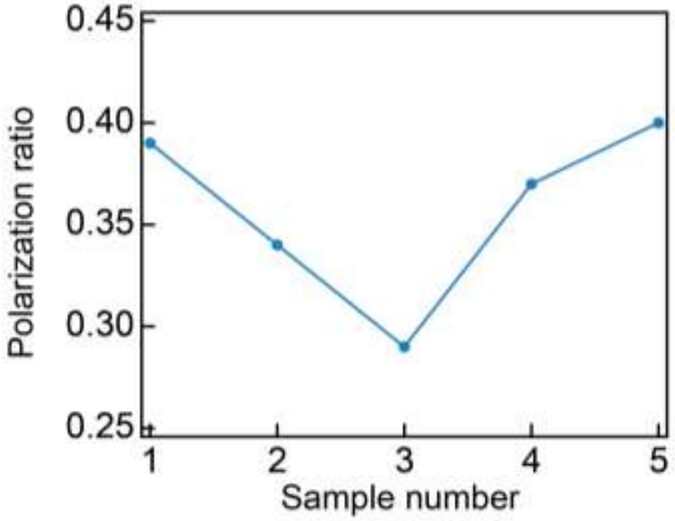

Figure S9. CP-SHG polarization ratio statistics from five different single crystalline samples of R-DPEDPb$_3$Br$_8$•H$_2$O.

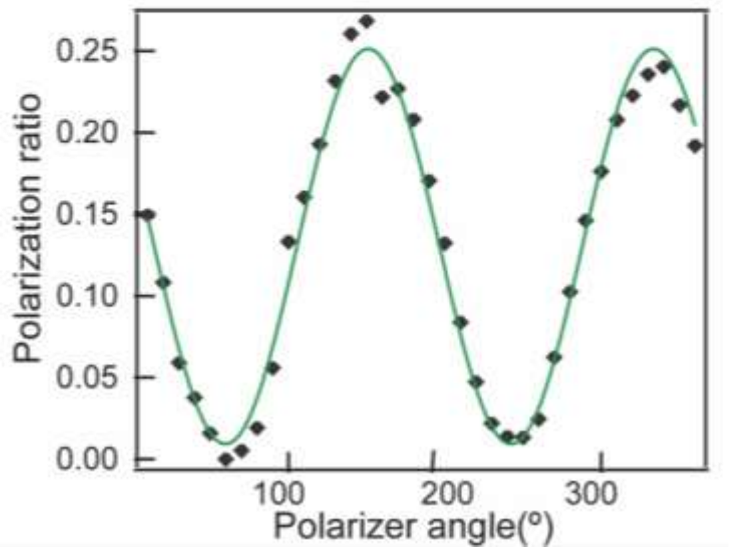

Figure S10. Polarization ratio of S-(DPED)Pb_3Br_8•H_2O as the function of azimuths angle of the linear polarizer.

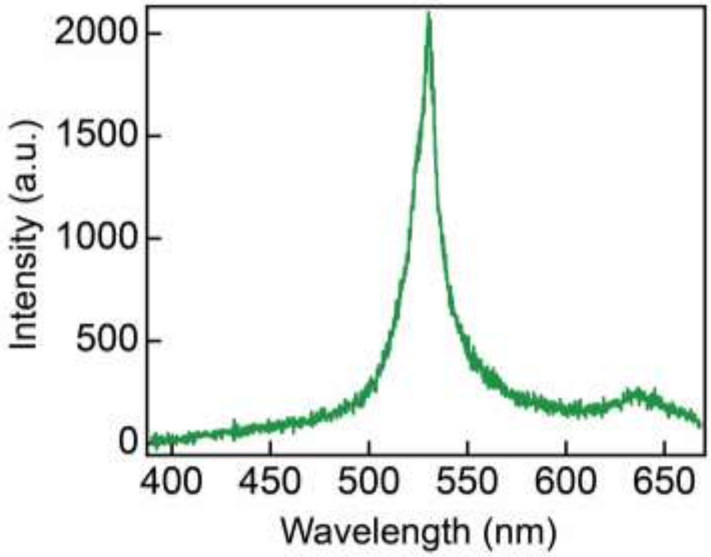

Figure S11. SHG spectrum of R-$DPEDPb_3Br_8$•H_2O thin film under 1060 nm excitation.

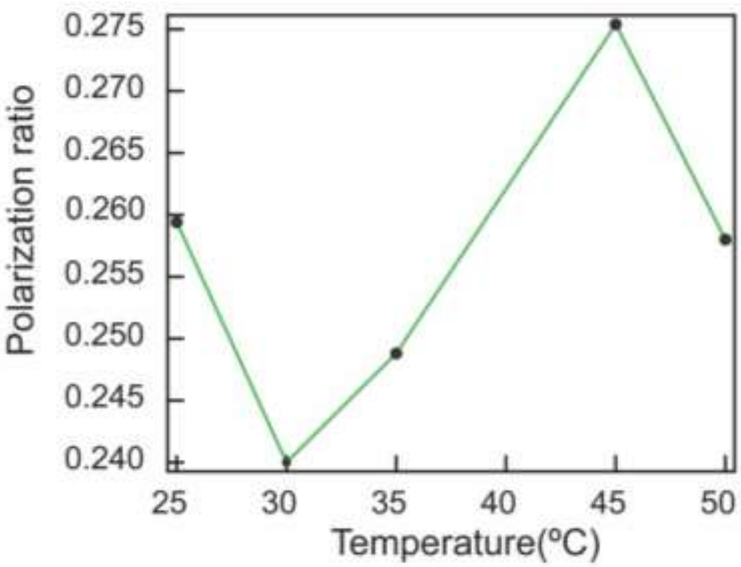

Figure S12. Temperature-dependent polarization ratio from CP-SHG of R-(DPED)Pb_3Br_8•H_2O.

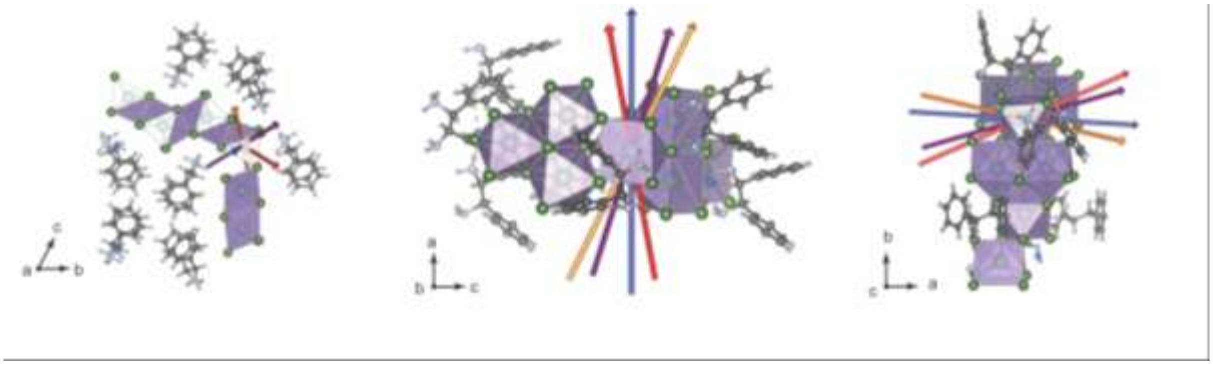

Figure S13. K vector diagram in crystal structure of R-(DPED)$Pb_3Br_8 \cdot H_2O$ viewing down the *a*-, *b*-, *c*-axis (Blue: Gamma to X, Purple: Gamma to R, Orange: Gamma to U, and Red: Gamma to V).

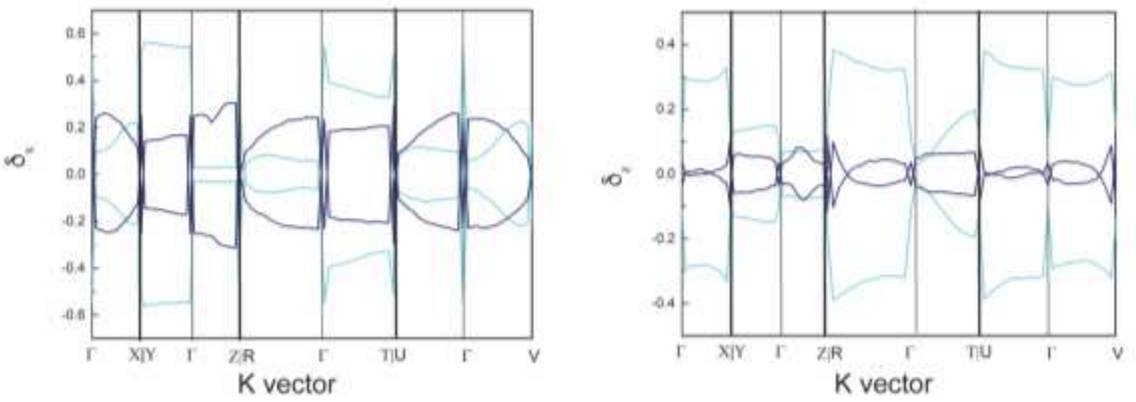

Figure S14. Momentum-dependent spin polarization from R-(DPED)Pb_3Br_8•H_2O along the x-axis (a) and z-axis (b) based on DFT calculations.
